# Rapid Increases in proBDNF after Pilocarpine-Induced Status Epilepticus in Mice Are Associated with Reduced proBDNF Cleavage Machinery^1^,^2^,^3^

**DOI:** 10.1523/ENEURO.0020-15.2016

**Published:** 2016-03-30

**Authors:** Ajay X. Thomas, Yasmin Cruz Del Angel, Marco I. Gonzalez, Andrew J. Carrel, Jessica Carlsen, Philip M. Lam, Barbara L. Hempstead, Shelley. J. Russek, Amy R. Brooks-Kayal

**Affiliations:** 1 Department of Pediatrics, University of Colorado Anschutz Medical Campus, Aurora, Colorado 80045; 2 Neuroscience Program, University of Colorado Anschutz Medical Campus, Aurora, Colorado 80045; 3 Medical Scientist Training Program, University of Colorado Anschutz Medical Campus, Aurora, Colorado 80045; 4 Departments of Medicine, Hematology & Medical Oncology, Weil Cornell Medical College, New York, New York 10045; 5 Department of Pharmacology, Boston University School of Medicine, Boston, Massachusetts 02118; 6 Department of Neurology, Children’s Hospital Colorado, Aurora, Colorado 80045

**Keywords:** BDNF, cleavage, epilepsy, PAI-1, ProBDNF, tPA

## Abstract

Brain-derived neurotrophic factor (BDNF) levels are elevated after status epilepticus (SE), leading to activation of multiple signaling pathways, including the janus kinase/signal transducer and activator of transcription pathway that mediates a decrease in GABA_A_ receptor α1 subunits in the hippocampus (Lund et al., 2008). While BDNF can signal via its pro or mature form, the relative contribution of these forms to signaling after SE is not fully known. In the current study, we investigate changes in proBDNF levels acutely after SE in C57BL/6J mice. In contrast to previous reports (Unsain et al., 2008; Volosin et al., 2008; VonDran et al., 2014), our studies found that levels of proBDNF in the hippocampus are markedly elevated as early as 3 h after SE onset and remain elevated for 7 d. Immunohistochemistry studies indicate that seizure-induced BDNF localizes to all hippocampal subfields, predominantly in principal neurons and also in astrocytes. Analysis of the proteolytic machinery that cleaves proBDNF to produce mature BDNF demonstrates that acutely after SE there is a decrease in tissue plasminogen activator and an increase in plasminogen activator inhibitor-1 (PAI-1), an inhibitor of extracellular and intracellular cleavage, which normalizes over the first week after SE. *In vitro* treatment of hippocampal slices from animals 24 h after SE with a PAI-1 inhibitor reduces proBDNF levels. These findings suggest that rapid proBDNF increases following SE are due in part to reduced cleavage, and that proBDNF may be part of the initial neurotrophin response driving intracellular signaling during the acute phase of epileptogenesis.

## Significance Statement

The studies reported here are the first to demonstrate acute changes in the expression of proBDNF within 3 h of the onset of status epilpticus (SE) that occur within principle cells and glia in all hippocampal subfields. We further found evidence that reduced expression of tissue plasminogen activator, part of the extracellular proteolytic cascade, and increased expression of plasminogen activator inhibitor-1, an inhibitor of extracellular and intracellular cleavage, may contribute to reduced proBDNF cleavage and elevations in proBDNF levels. These findings suggest that proBDNF may be part of the initial neurotrophin response driving intracellular signaling acutely after SE and during the earliest phase of epileptogenesis.

## Introduction

**Figure 1.**
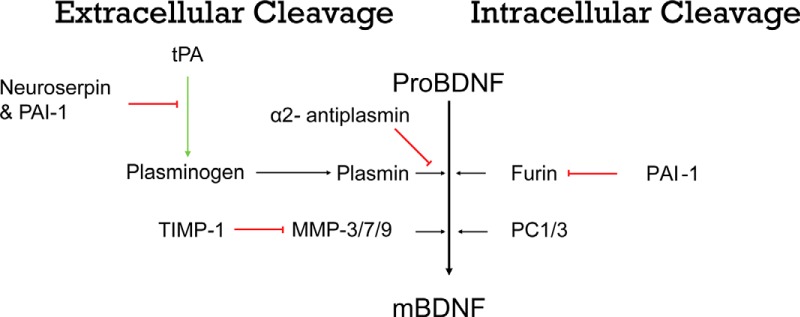
Schematic representation of different proteins involved in the cleavage of BDNF through extracellular (left panels) and intracellular (right panels) mechanisms. ProBDNF can be cleaved intracellularly within the endoplasmic reticulum by furin and in regulated secretory vesicles by proconvertase enzymes (PC1/3). ProBDNF can also be cleaved extracellularly by MMPs (−3/−7/−9) or by components of the tPA/plasmin proteolytic cascade. The activity of these proteases is tightly regulated by a number of inhibitors, including PAI-1, which inhibits both extracellular and intracellular cleavage; TIMPs, which inhibit MMPs; and neuroserpin and A2AP, which inhibit the tPA/plasmin proteolytic cascade. Red bars indicate inhibition, and green bars indicate activation.

Brain-derived neurotrophic factor (BDNF) promotes growth and differentiation of neurons during development and plays an important role in many physiological processes, such as learning and memory, as well as various pathological processes, such as epileptogenesis (Lu et al., 2014). Synthesis and expression of BDNF are highly regulated throughout the nervous system (Lessmann and Brigadski, 2009). BDNF is initially synthesized as a precursor protein (preproBDNF) in the endoplasmic reticulum and is transported to the Golgi as proBDNF once the signal peptide is cleaved. Mature BDNF (mBDNF) can be produced intracellularly by furin-mediated cleavage or by proprotein convertase in immature secretory granules (Mowla et al., 1999). ProBDNF can also be cleaved extracellularly by matrix metalloproteinases (MMPs; −3, −7, or 9), or by components of the tissue plasminogen activator/plasmin (tPA/plasmin) proteolytic cascade (Lee et al., 2001; Pang et al., 2004). The activity of these proteases is tightly regulated. Plasminogen activator inhibitor-1 (PAI-1) inhibits both tPA and furin, inhibiting both extracellular and intracellular cleavage (Binder et al., 2002; Dupont et al., 2009; Bernot et al., 2011; Fig. 1). In addition, tissue inhibitor of metalloproteinases (TIMPs) inhibit MMPs, while neuroserpin and α2 antiplasmin (A2AP) inhibit the tPA/plasmin proteolytic cascade (Hastings et al., 1997; Krueger et al., 1997; Brew et al., 2000; Yepes and Lawrence, 2004; Coughlin, 2005).

Several studies have demonstrated that a significant portion of BDNF protein is secreted as proBDNF and cleaved extracellularly via the tPA/plasmin proteolytic cascade (Pang et al., 2004; Nagappan et al., 2009). *In vitro*, high-frequency neuronal activity triggers the simultaneous release of proBDNF and tPA to generate mBDNF extracellularly (Nagappan et al., 2009), suggesting that this could occur *in vivo* after the repeated neuronal firing that is observed during seizures. However, the *in vivo* effects of acute seizures on proBDNF levels have not yet been fully elucidated.

Numerous reports suggest that BDNF levels are increased in the hippocampus after seizures induced by kindling (Ernfors et al., 1991), electroconvulsive shock (Altar et al., 2004), kainate (Rudge et al., 1998), and pilocarpine (Roberts et al., 2006). In addition, several studies suggest a pro-epileptogenic effect of BDNF that appears to be mediated at least in part by activation of the tropomyosin-receptor kinase B (TrkB) receptors (McNamara et al., 2006). However, other studies suggest that intrahippocampal infusion of BDNF results in enhanced resistance to kindling and may protect against epileptogenesis (Larmet et al., 1995; Reibel et al., 2000). These contrasting findings may be due, in part, to differential actions of proBDNF and mBDNF during epileptogenesis.

A potential role for the proneurotrophins in epileptogenesis is starting to emerge. Enhancing cleavage of pro-nerve growth factor (proNGF) to generate mature NGF provides neuroprotection after the administration of kainate to organotypic slice cultures (Le and Friedman, 2012). In rodents, increases in BDNF mRNA occur as early as 3 h after pilocarpine-induced status epilepticus (SE; Mudò et al., 1996), and increased proBDNF has been detected 24 h after SE induction (Volosin et al., 2008; VonDran et al., 2014). More recently, it has been reported that high-dose proBDNF applied to cultured hippocampal neurons may cause alterations in GABAergic neurotransmission by promoting GABA_A_ receptor (GABA_A_R) endocytosis and degradation through activation of the RhoA–Rock–PTEN (phosphatase and tensin homolog) pathway, and may contribute to repression of GABA_A_R synthesis through activation of the janus kinase/signal transducer and activator of transcription (JAK/STAT) pathway (Riffault et al., 2014). The addition of exogenous BDNF to neuronal cultures rapidly increases STAT3 phosphorylation (Ng et al., 2006; Lund et al., 2008). BDNF-dependent activation of the JAK/STAT pathway in rat dentate gyrus occurs within 6 h of SE onset and drives a decrease in mRNA for the α1 subunit of GABA_A_R (Lund et al., 2008; Grabenstatter et al., 2014), suggesting that BDNF-induced activation of the JAK/STAT pathway occurs rapidly after SE onset.

To better understand the potential contribution of proBDNF during the earliest phases of epileptogenesis, we used proBDNF-specific antibodies in wild-type (WT) C57BL/6J mice and knock-in mice on a C57BL/6J background that express a hemagglutinin-tagged *bdnf* transgene under the control of endogenous *bdnf* promoters (Yang et al., 2009) to assess the levels and localization of BDNF acutely following the induction of pilocarpine-induced SE. The study finds that within the first 3 h after SE onset there is an acute increase in proBDNF levels in principal neurons and glia in all hippocampal subfields, as well as altered expression of both tPA and PAI-1 that would be predicted to reduce proBDNF cleavage. Together, these data suggest that reduced BDNF cleavage acutely after SE leads to proBDNF accumulation, which may be the initial neurotrophin driving cell signaling during early epileptogenesis.

## Materials and Methods

### Induction of SE

All animal procedures were performed in accordance with the regulations of the institutional animal care and use committees of the University of Colorado Anschutz Medical Campus, Weil Cornell Medical College, Boston University Medical School, and Children’s Hospital Colorado; and the National Institutes of Health *Guide for the Care and Use of Laboratory Animals*. Adult male animals were used for all studies, and were group housed with up to five age-matched littermates in temperature- and humidity-controlled rooms with access to food and water *ad libitum* on a 12 h light/dark cycle.

Knock-in mice that express a *bdnf* allele with a hemagglutinin tag added to the C terminus of the murine coding exon of BDNF (BDNF-HA) were generously provided by the Hempstead group (Weill Cornell Medical College; Yang et al., 2009). BDNF-HA mice were backcrossed on a C57BL/6J background for >10 generations. WT C57BL/6J mice (The Jackson Laboratory) were used for studies with proBDNF antibody detection and the protease machinery studies. SE was induced using repetitive intraperitoneal injections of pilocarpine, as previously described (Müller et al., 2009). The mice acquired from The Jackson Laboratory were received at 5 weeks of age and were allowed to rest for 1 week prior to handling in order to acclimate to the environment and altitude. Six- to 8-week-old WT and BDNF-HA mice (18-24 g) were handled for at least 1 week to reduce the stress produced by handling required for the induction of SE. The induction protocol was initiated between 7:00 and 8:00 A.M. to minimize diurnal variation. The mice were transferred to the induction room, marked, weighed, and allowed to rest undisturbed for at least 1 h. To block the peripheral muscarinic effects of pilocarpine, each mouse was given an intraperitoneal injection of 1 mg/kg scopolamine methyl bromide (Sigma-Aldrich) 15 min before the first pilocarpine injection on the day of seizure induction. An initial dose of pilocarpine HCl (200 mg/kg; Sigma-Aldrich) was given, then 1 h after the first injection subsequent doses (100 mg/kg) were given at 30 min intervals. The animals were group housed with up to five age-matched littermates and then separated into individual cages after the third injection for individual monitoring of behavioral seizures. Injections were discontinued at the onset of SE, which was defined by the appearance of repeated behavioral seizures (stage four or higher with at least one seizure being five or higher) according to a modified Racine scale (Borges et al., 2003).

SE typically initiated approximately 3 h after the first injection, requiring at least three injections (400 mg/kg total dose) of pilocarpine with an average of five injections (600 mg/kg total dose) of pilocarpine. SE persisted for at least 90 min with ∼30% of animals successfully undergoing SE and surviving until their respective time points. The specific cause of death cannot be definitively determined, but postconvulsion respiratory failure appeared to be a common cause of acute death after pilocarpine administration (as has been previously described; Boyd and Fulford, 1961). Control mice were given injections of saline at identical time intervals. Mice that were killed >3 h after SE induction were returned to their housing room and given free access to water, Gatorade, and moistened chow with equal parts sucrose. Mice were killed with rapid isoflurane-induced anesthesia followed by decapitation. Fresh tissue for Western blot was collected via rapid hippocampal dissection in ice-cold PBS containing phosphatase inhibitors (phosphatase inhibitor cocktail 2, P5726, Sigma-Aldrich) and frozen on dry ice. The two hippocampi from each animal were pooled into a single sample for that animal, and samples were stored at −80°C until lysate preparation. For immunohistochemistry, mice were killed at 3 h after SE onset by deep anesthesia with ketamine/xylazine and inhaled isoflurane followed by rapid intracardiac perfusion with ice-cold PBS then 4% paraformaldehyde in phosphate buffer at pH 7.4. The brains were dissected out, postfixed overnight in 4% paraformaldehyde, and underwent cryoprotection in 30% sucrose in PBS; and were then stored at −80°C in Tissue-Tek O.C.T. Compound (Sakura Finetek) until sectioning.

### Western blotting

The frozen hippocampi were lysed in RIPA buffer (50 mm Tris-HCl, pH 7.4, 150 mm NaCl, 0.25% deoxycholic acid, 1% NP-40, and 1 mm EDTA) with 10 mm phenylmethylsulfonyl fluoride, 10 mm sodium orthovanadate, 10 mm sodium fluoride, phosphatase inhibitor cocktail 2 (1:250), and protease inhibitor cocktail (1:250; P8340, Sigma-Aldrich) using an ultrasonic sonifier. Samples were then gently shaken at 4°C for 30 min and centrifuged at 14,000 × *g* for 30 min at 4°C. The supernatants were reserved, aliquoted, and stored at −80°C until SDS-PAGE. Identical amounts of protein were loaded per lane for each sample on all blots probed with a given antibody, with 20-60 μg of protein used depending on the specific antibody used. After gel transfer, the nitrocellulose membranes were blocked with 5% milk (furin and MMP-9 were blocked with 5% nonfat dry milk, 2% BSA, 4% FBS, 4% normal horse serum, and 4% normal goat serum). The blots probed with the anti-HA and BDNF antibody were first washed with Tris-buffered saline–Tween-20 (TBST; 50 mm Tris-Base, 150 mm NaCl, 0.05% Tween-20, pH 7.6), and then were fixed with 2.5% glutaraldehyde in PBS, washed twice with PBS, washed twice with TBST, and then blocked with 5% milk in TBST. All membranes were incubated overnight at 4°C with their respective primary antibody in diluted blocking buffer. The following antibodies and concentrations were used: mouse monoclonal HA.11 clone 16B12 antibody (1:3000; MMS-101P, Covance), mouse monoclonal proBDNF antibody (1:1000; H10001G-MA, GeneCopoeia), rabbit polyclonal to α-2 antiplasmin (1:2000; ab62771, Abcam), rabbit polyclonal furin antibody (1:1000; sc-20801, Santa Cruz Biotechnology), rabbit polyclonal MMP-9 antibody (1:2000; AB13458, Millipore), sheep polyclonal neuroserpin antibody (1:2000; SASMNSP-GF-HT, Molecular Innovations), rabbit polyclonal PAI-1 antibody (1:1000; ASMPAI-GF-HT, Molecular Innovations), rabbit polyclonal plasminogen antibody (1:3000; ASMPLG-GF-HT, Molecular Innovations), sheep polyclonal tPA antibody (1:500; SASTPA-GF-HT, Molecular Innovations), and rabbit polyclonal TIMP-1 antibody (1:1000; AB770, Millipore). Following incubation with the appropriate secondary antibody, membranes were incubated with SuperSignal West Dura Chemiluminescent Substrate (Pierce) with the anti-HA blots being enhanced with Lumigen TMA-6 (LUMIGEN). Blots were stripped with 50 mm glycine, pH 2.3, and reprobed with other primary antibodies or actin (1:20,000–80,000; A2066, Sigma-Aldrich). The Western blotting results presented include representative images of the blots run in duplicate and adjusted for contrast, and the densitometry quantitation of each band normalized to actin that was used as a loading control to ensure consistent protein amounts were loaded across samples using FIJI (Schindelin et al., 2012). The average of the normalized densitometry measurements for the control group was considered 100%, with error bars reported as SEM and *N* referring to the number of samples consisting of lysates from both hippocampi from individual animals in each group.

## Immunohistochemistry

Brains were sectioned at 30 μm into cryoprotectant (30% sucrose, 30% ethylene glycol, and 0.1 m phosphate buffer) and stored at −20°C for floating section staining. Sections were washed in PBS several times; then blocked with 3% BSA, 3% normal goat serum, and 3% normal donkey serum with 0.1% Triton X-100 in PBS for 1 h at room temperature; and then incubated overnight at 4°C with rabbit polyclonal HA antibody (1:500; A6908, Sigma-Aldrich) in combination with chicken polyclonal microtubule-associated protein 2 (MAP2) antibody (1:1000; ab5392, Abcam) and guinea pig polyclonal GFAP antibody (1:500; 174004, Synaptic Systems). After primary antibody washing, sections were incubated for 1 h with a biotinylated goat anti-rabbit IgG secondary antibody (1:400; 111-065-144, Jackson ImmunoResearch), a goat anti-chicken IgY Alexa Fluor 568, and donkey anti-guinea pig IgG Alexa Fluor 647 to detect MAP2 and GFAP, respectively. Sections were subsequently incubated for 1 h with Alexa Fluor 488-streptavidin (1:800; 016-580-084, Jackson ImmunoResearch) to visualize the HA tag. The sections were mounted on glass slides with VECTASHIELD mounting medium with DAPI (Vector Laboratories), coverslipped, and sealed.

### Confocal microscopy

Slide-mounted sections of immunolabeled hippocampi were viewed on an inverted microscope (Axio Examiner Z1, Carl Zeiss) equipped with Plan-Apochromat 20× [0.8 numerical aperture (NA)] or 63× (oil differential interference contrast; 1.4 NA) objectives and attached to a spectral confocal laser system (LSM 780, Carl Zeiss) powered by ZEN 2012 software (Carl Zeiss). The tissue was scanned at room temperature with a tunable infrared Coherent Chameleon Ultra II laser tuned to 800 nm to detect DAPI staining and 488, 561, and 633 nm laser lines to detect the Alexa fluorophores 488, 568, and 647, respectively. Images were acquired as *z*-stacks using sequential line (mean of four) scanning. Colocalization of two fluorophores with DAPI was simultaneously qualitatively assessed in the *x*-, *y*-, and *z*-planes of each optical section. Average projection images of five optical slices every 2 μm on the *z*-axis were produced, and minimal adjustments to image contrast and intensity were made in FIJI (Schindelin et al., 2012) using the levels or contrast/brightness functions. All brains and sections were processed in parallel with images acquired, adjusted, and analyzed in an identical manner between SE and control animals. Images were arranged and annotated using Illustrator (Adobe).

## Acute hippocampal section studies

Animals were anesthetized with isoflurane, killed, the brain swiftly removed, and placed in cold (4°C) oxygenated (95% O_2_, 5% CO_2_) sucrose-modified artificial CSF (aCSF) containing the following (in mm): sucrose 45, NaCl 87, glucose 25, NaHCO_3_ 25, KCl 2.5, NaH_2_PO_4_ 1.25, MgCl_2_ 7, and CaCl_2_ 0.5, pH 7.4 and 300–310 mOsm. Transverse hippocampal slices (300 μm) were obtained using a slicing vibratome (VT1200s, Leica). Hippocampi were dissected out from slices in the cold sucrose-modified aCSF solution and rinsed briefly in oxygenated aCSF containing the following (in mm): NaCl 130, glucose 10, NaHCO_3_ 25, KCl 3.5, NaH_2_PO_4_ 1.25, MgCl_2_ 2, CaCl_2_ 2, pH 7.4 and 300–305 mOsm. Rinsed slices were then placed on ice in a 6-well plate with 5 ml of aCSF in individual wells modified to allow the delivery of oxygen. Alternating slices from the two hippocampi from a single animal were placed in wells for the vehicle or PAI-1 inhibitor group, and care was taken to assure that a similar number of sections from each hippocampus was placed in each well. Once sectioning was completed, 25 μl of vehicle (DMSO, Sigma-Aldrich) was added to one of the wells (vehicle group), and 25 μl of the PAI-1 inhibitor tiplaxtinin (Axon 1383; 74 mm stock in DMSO) was added to the other well (final concentration, 370 μm; Axon Medchem). Both the vehicle and inhibitor wells were carefully mixed with repeated aspiration to obtain equal distribution in the aCSF. Once the vehicle and inhibitor were added, the 6-well plate was placed into a water bath and incubated at 36°C for 4 h with constant, low-pressure oxygenation. After incubation, slices were collected, flash frozen using dry ice, and stored at −80°C until lysate preparation.

## Results

**Figure 2.**
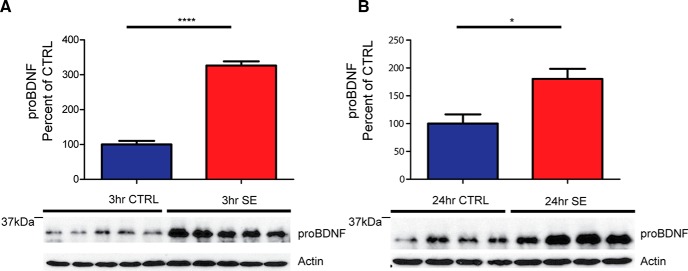
ProBDNF protein levels are elevated acutely after pilocarpine-induced SE in WT C57BL/6J mice. ***A***, Bottom, Representative Western blot of whole hippocampal protein homogenates from WT mice killed 3 h after the induction of SE or time-matched saline controls probed with proBDNF (1:1000) and anti-actin antibodies. Top, Densitometry analysis of proBDNF protein abundance. Ratio of proBDNF/actin at 3 h after SE (*N* = 5), expressed as the percentage change relative to mean values (±SEM) of the control group (*N* = 5; ****p < 0.001). ***B***, Bottom, Representative Western blot of whole hippocampal protein homogenates from WT mice killed 24 h after the induction of SE or time-matched saline controls probed with proBDNF (1:1000) and anti-actin antibodies. Top, Ratio of proBDNF/actin at 24 h after SE (*N* = 4) expressed as the percentage change relative to mean values of the control group (*N* = 4; *p < 0.05).

Levels of proBDNF protein expression were initially assessed in whole hippocampal lysates from age-matched pilocarpine- and saline-treated WT C57BL/6J mice using a mouse monoclonal antibody specific for proBDNF (GeneCopoeia). A significant increase in the immunoreactivity of proBDNF was observed at 3 h [Fig. 2*A*; control, 100.0 ± 10.6 (*N* = 5) vs SE, 326.4 ± 12.2 (*N* = 5); *t* test, *p* < 0.001] and 24 h [Fig. 2*B*; 100.0 ± 16.6 (*N* = 4) vs 180.4 ± 18.0 (*N* = 4); *t* test, *p* < 0.05] after SE onset (Table 1). These data suggest that a significant increase in proBDNF protein levels occurs as soon as 3 h after pilocarpine-induced SE.

**Table 1:** Statistics

	Data structure	Type of test	Power
Figure 2A Increased proBDNF 3 h post-SE (HA immunoreactivity)	Normal distribution	Student’s *t* test	0.9775
Figure 2B Increased proBDNF 24 h post-SE (HA immunoreactivity)	Normal distribution	Student’s *t* test	0.9917
Figure 3A Increased proBDNF 3 h post-SE (commercial BDNF antibodies)	Normal distribution	Student’s *t* test	1.0000
Figure 3B Increased proBDNF 24 h post-SE (commercial BDNF antibodies)	Normal distribution	Student’s *t* test	0.9104
Figure 5A No change in furin 3 h post-SE	Normal distribution	Student’s *t* test	0.0511
Figure 5B Significant increase in furin 24 h post-SE	Normal distribution	Student’s *t* test	0.9198
Figure 5C No change in plasminogen 3 h post-SE	Normal distribution	Student’s *t* test	0.5067
Figure 5D No change in plasminogen 24 h post-SE	Normal distribution	Student’s *t* test	0.6526
Figure 5E No change in MMP-9 3 h post-SE	Normal distribution	Student’s *t* test	0.1905
Figure 5F No change in MMP-9 24 h post-SE	Normal distribution	Student’s *t* test	0.2765
Figure 5G Significant reduction in tPA 3 h post-SE	Normal distribution	Student’s *t* test	0.9394
Figure 5H Significant reduction in tPA 24 h post-SE	Normal distribution	Student’s *t* test	1.0000
Figure 6A No change in A2AP 3 h post-SE	Normal distribution	Student’s *t* test	0.5646
Figure 6B No change in A2AP 24 h post-SE	Normal distribution	Student’s *t* test	0.1068
Figure 6C Reduction in neuroserpin at 3 h post-SE	Normal distribution	Student’s *t* test	0.9961
Figure 6D No change in neuroserpin at 24 h post-SE	Normal distribution	Student’s *t* test	0.0744
Figure 6E No change in 23 kDa nonglycosylated TIMP-1 at 3 h post-SE	Normal distribution	Student’s *t* test	0.1867
Figure 6E No change in 28 kDa glycosylated TIMP-1 at 3 h post-SE	Normal distribution	Student’s *t* test	0.5007
Figure 6F No change in 23 kDa nonglycosylated TIMP-1 at 24 h post-SE	Normal distribution	Student’s *t* test	0.6078
Figure 6F Significant reduction in 28 kDa glycosylated TIMP-1 at 24 h post-SE	Normal distribution	Student’s *t* test	0.9987
Figure 6G Significant increase in PAI-1 at 3 h post-SE	Normal distribution	Student’s *t* test	0.9601
Figure 6H Significant increase in PAI-1 at 24 h post-SE	Normal distribution	Student’s *t* test	1.0000
Figure 7A Increased proBDNF 3 d post-SE (commercial BDNF antibodies)	Normal distribution	Student’s *t* test	0.971
Figure 7A Increased proBDNF 7 d post-SE (commercial BDNF antibodies)	Normal distribution	Student’s *t* test	1.000
Figure 7B Increased PAI-1 3 d post-SE (commercial BDNF antibodies)	Non-normal distribution	Mann–Whitney test	N/A
Figure 7B No change in PAI-1 7 d post-SE (commercial BDNF antibodies)	Normal distribution	Student’s *t* test	0.985
Figure 7C No change in tPA 3 d post-SE (commercial BDNF antibodies)	Normal distribution	Student’s *t* test	0.981
Figure 7C Increased tPA 7 d post-SE (commercial BDNF antibodies)	Normal distribution	Student’s *t* test	1.000
Figure 8 PAI-1 inhibition reduces proBDNF levels after pilocarpine SE	N/A	Paired *t* test	0.995

**Figure 3.**
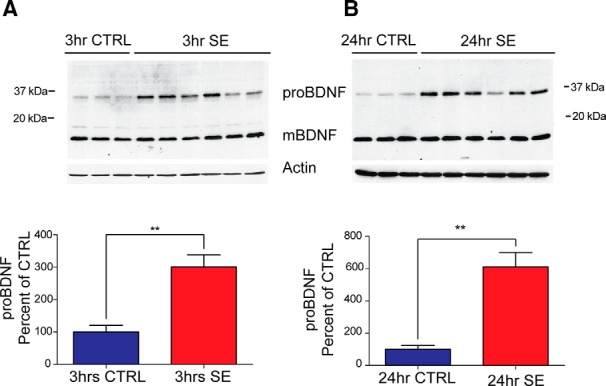
ProBDNF levels are elevated in BDNF-HA-tagged mice in the first 24 h after pilocarpine-induced SE. ***A***, Top, Representative Western blot of whole hippocampal protein homogenates from BDNF-HA mice killed 3 h after the induction of SE or time-matched saline controls probed with anti-HA (1:3000) and anti-actin antibodies. Bottom, Densitometry analysis of proBDNF protein abundance. Ratio of proBDNF/actin at 3 h after SE (*N* = 6) expressed as the percentage change relative to mean values (±SEM) of the control group (*N* = 3; **p < 0.001). ***B***, Top, Representative Western blot of whole hippocampal protein homogenates from BDNF-HA mice probed with anti-HA (1:3000) and anti-actin antibodies killed 24 h after the induction of SE or time-matched saline controls. Bottom, Densitometry analysis of proBDNF protein abundance at 24 h after SE. Ratio of proBDNF/actin at 24 h post-SE (*N* = 6) expressed as the percentage change relative to mean values of control group (*N* = 3; **p < 0.01). Densitometry analysis of mBDNF protein abundance (mBDNF/actin) showed no significant difference between the control and SE group at either time point.

To further assess proBDNF levels and cellular localization, we used knock-in mice that express a *bdnf* allele with BDNF-HA. Tissue from control BDNF-HA mice (controls) or BDNF-HA mice subjected to pilocarpine-induced SE was collected at 3 and 24 h after SE onset. Western blot analysis showed a significant increase in proBDNF corresponding to a 34 kDa HA-immunoreactive band as early as 3 h after SE induction [Fig. 3*A*; control, 100.0 ± 20.5 (*N* = 3) vs SE, 300.4 ± 37.4 (*N* = 6); *t* test, *p* < 0.01]. In addition, a significant increase in proBDNF immunoreactivity was also observed 24 h after SE [Fig. 3*B*; control, 100.0 ± 29.3 (*N* = 3) vs SE, 610.1 ± 89.7 (*N* = 6); *t* test, *p* < 0.01]. In contrast, no difference in HA immunoreactivity was observed for the 14 kDa band corresponding to mBDNF between SE and control animals at either time point [Fig. 3*A*,*B*; 3 h: control, 100.0 ±12.2 (*N* = 3) vs SE, 96.4 ± 5.8 (*N* = 6); 24 h: control, 100.0 ± 4.9 (*N* = 3) vs SE, 98.5 ± 5.0 (*N* = 6); *t* test, *p* > 0.05 at both time points]. Together, these data provide further evidence that a significant increase in the levels of proBDNF are triggered acutely after SE onset.

**Figure 4.**
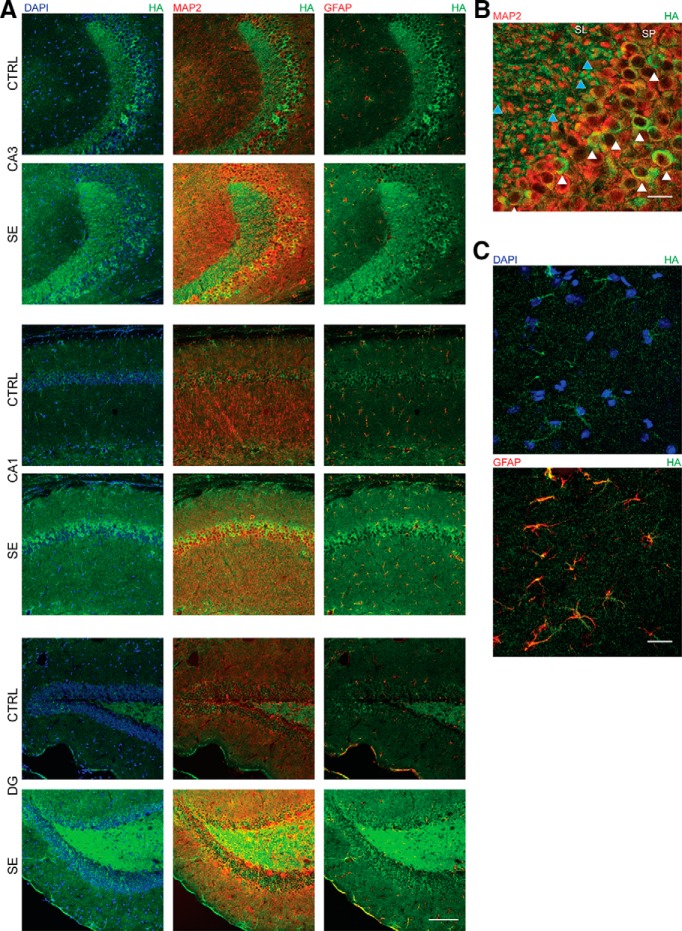
BDNF protein is expressed in neurons and astrocytes of hippocampus after pilocarpine-induced SE. ***A***, Representative confocal images of hippocampal subfields from HA-tagged mice 3 h after SE and an age- and handling-matched control (20× magnification; scale bar, 100 µm) shows the presence of HA immunoreactivity in principal cells, glia, and mossy fiber layers. The first column shows anti-HA (green) immunoreactivity with DAPI (blue) in each condition. The second column demonstrates the colocalization of immunoreactivity for HA (green) and the neuronal marker MAP2 (red). The third column demonstrates colocalization of immunoreactivity for HA (green) and the glial marker GFAP (red). ***B***, High-magnification confocal image of CA3 hippocampal subfield (63× magnification; scale bar, 20 µm). White arrowheads correspond to neuronal localization of HA immunoreactivity in pyramidal cells of CA3; blue arrowheads correspond to the localization of HA immunoreactivity in mossy fibers. SL, Stratum lucidum; SP, stratum pyramidale. ***C***, High-magnification confocal image of CA3 hippocampal subfield (63× magnification; scale bar, 20 µm), demonstrating glial expression of BDNF.

To determine the cellular distribution of BDNF protein after SE, HA immunoreactivity was analyzed in coronal sections colabeled with a neuronal (MAP2) and an astrocytic (GFAP) marker (Fig. 4). It is important to note that the HA immunostaining is unable to distinguish between proBDNF and mBDNF; therefore, in these experiments the signal detected was considered as total BDNF expression. Another important limitation of these findings is that one cannot distinguish between HA immunoreactivity indicating the site of BDNF prerelease or internalization. Colocalization of HA and MAP2 immunoreactivity was assessed to identify BDNF expression in neurons and colocalization of HA and GFAP immunoreactivity was assessed to identify BDNF expression in astrocytes. The observed pattern of protein expression detected in control animals is consistent with the pattern of expression previously reported by others using the same HA-tagged BDNF knock-in mice (Yang et al., 2009; Dieni et al., 2012). The most prominent HA immunoreactivity signal is detected in the mossy fiber pathway, CA3 pyramidal neurons, and CA1 pyramidal neurons (Fig. 4). When compared with controls, mice at 3 h post-SE showed an increase in HA immunoreactivity as well as MAP2 immunoreactivity that can be detected in all hippocampal regions analyzed. In SE animals, the pattern of HA immunoreactivity is well colocalized with both MAP2 (Fig. 4*A*,*B*) and GFAP immunostaining (Fig. 4*A*,*C*), suggesting that within the hippocampus of animals acutely following SE, BDNF is localized in principal neurons and astrocytes.

**Figure 5.**
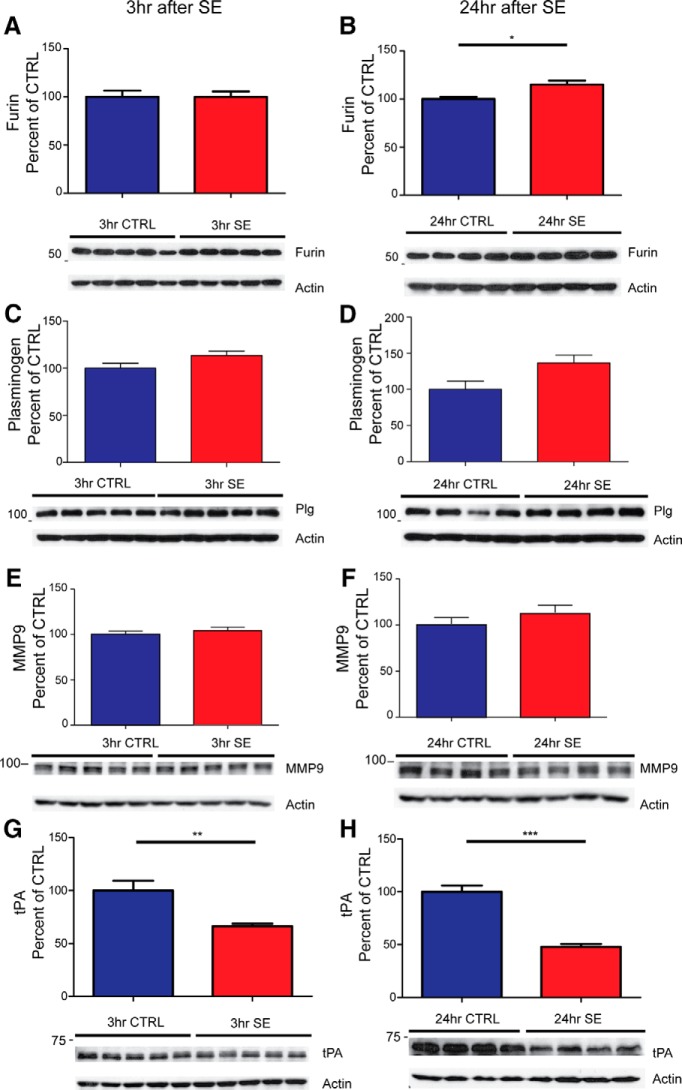
Enzymes involved in the processing of proBDNF are altered after pilocarpine-induced SE. Representative Western blots of whole hippocampal protein homogenates from WT mice killed 3 h (left panels) and 24 h (right panels) after the induction of SE or time-matched saline controls. Densitometry analysis of abundance of different cleavage proteins normalized to actin and expressed as the percentage change relative to mean values of the control group (±SEM). ***A***–***H***, Anti-furin (1:1000; ***A***, ***B***); anti-plasminogen (1:3000; ***C***, ***D***); anti-MMP9 (1:2000; ***E***, ***F***); anti-tPA (1:1000; ***G***, ***H***). The sample size for 3 h is *N* = 5 in each group and for 24 h is *N* = 4 in each group. *p < 0.05, **p < 0.01, ***p < 0.001; *t* test.

The very rapid elevation of proBDNF levels detected by Western blot suggests that in addition to the previously documented increase in BDNF expression, the typically rapid cleavage of proBDNF might also be impaired after SE. Therefore, the expression of enzymes involved in proBDNF cleavage were analyzed via Western blot (*n* = 5 for each group at 3 h, and *n* = 4 for each group at 24 h for all assays). Our analyses showed that there is no statistical difference in furin immunoreactivity 3 h after SE (Fig. 5*A*; control, 100.0 ± 6.5 vs SE, 99.9 ± 5.7; *t* test, *p* > 0.05), while there is a modest but significant increase in furin expression at 24 h after SE (Fig. 5*B*; control, 100.0 ± 2.202 vs SE, 115.1 ± 3.970; *t* test, *p* < 0.05). In contrast, there is no statistical difference in the immunoreactivity of plasminogen observed at 3 h after SE (Fig. 5*C*; control, 100.0 ± 5. 4 vs SE, 113.4 ± 5.0; *t* test, *p* > 0.05) or 24 h after SE (Fig. 5*D*; control, 100.0 ± 11.76 vs SE, 136.8 ± 10.8; *t* test, *p* > 0.05). In addition, there is no statistical difference in the immunoreactivity of MMP-9 observed at 3 h after SE (Fig. 5*E*; control, 100.0 ± 3.7 vs SE, 104.4 ± 3.7; *t* test, *p* > 0.05) or 24 h after SE (Fig. 5*F*; control, 100.0 ± 6.9 vs SE, 112.5 ± 8.0; *t* test, *p* > 0.05). Interestingly, a significant decrease in tPA expression was observed at both 3 h after SE (Fig. 5*G*; control, 100.0 ± 9.3 vs SE, 66.3 ± 2.4; *t* test, *p* < 0.01) and 24 h after SE (Fig. 5*H*; control, 100.0 ± 5.9 vs SE, 47.9 ± 2.7; *t* test, *p* < 0.001).

**Figure 6.**
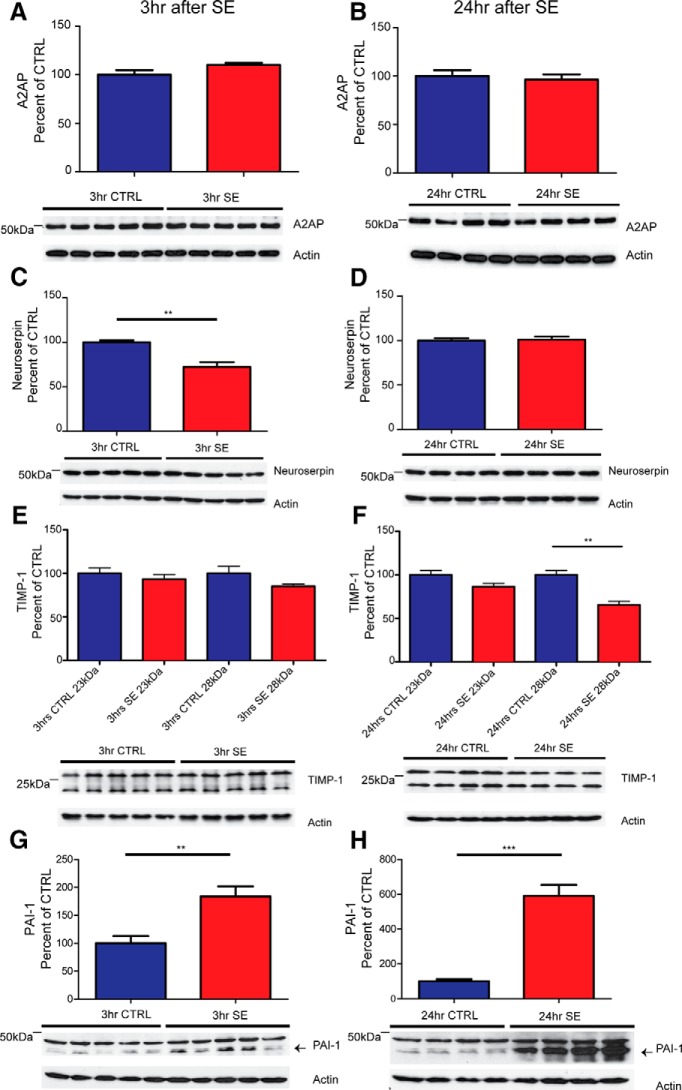
Inhibitors of proBDNF processing are altered after pilocarpine SE. Representative Western blots of whole hippocampal protein homogenates from WT mice killed 3 h (left panels) and 24 h (right panels) after the induction of SE or time-matched saline controls. Densitometry analysis of abundance of different inhibitor proteins normalized to actin and expressed as the percentage change relative to mean values of the control group (±SEM). ***A–H***, Anti-A2AP (1:2000; ***A***, ***B***); anti-neuroserpin (1:2000; ***C***, ***D***); anti-TIMP-1 (1:1000; ***E***, ***F***); and anti-PAI-1 (1:1000; ***G***, ***H***). The sample size for 3 h is *N* = 5 in each group, and for 24 h it is *N* = 4 in each group. **p < 0.01, ***p < 0.001; *t* test.

In order to further investigate the mechanism of proBDNF cleavage after SE, the levels of protease inhibitors known to inhibit the activity of proBDNF cleavage enzymes were analyzed. We observed no change in the immunoreactivity of A2AP, an inhibitor of plasmin activity, at 3 h after SE (Fig. 6*A*; control, 100.0 ± 4.8 vs SE, 110.2 ± 1.9; *t* test, *p* > 0.05) or 24 h after SE (Fig. 6*B*; control, 100.0 ± 6.0 vs SE, 96.4 ± 5.3; *t* test, *p* > 0.05). A slight reduction in the immunoreactivity of neuroserpin, a known inhibitor of tPA activity, was observed at 3 h after SE (Fig. 6*C*; control, 100.0 ± 2.2 vs SE, 72.3 ± 5.4; *t* test, *p* < 0.01) but not at 24 h after SE (Fig. 6*D*; control, 100.0 ± 2.7 vs SE, 101.0 ± 3.6; *t* test, *p* > 0.05). As seen in Figure 6, *E* and *F*, two forms of TIMP-1, an inhibitor of MMP activity, can be detected via Western blot, a 23 kDa nonglycosylated and a 28 kDa glycosylated form. There is no statistical difference in the immunoreactivity of the nonglycosylated form at 3 h after SE (Fig. 6*E*; control, 100.0 ± 6.1 vs SE, 93.3 ± 5.4; *t* test, *p* > 0.05) or 24 h after SE (Fig. 6*F*; control, 100.0 ± 4.9 vs SE, 86.41 ± 3.9; *t* test, *p* > 0.05). In addition, there is no statistical difference in the immunoreactivity of the glycosylated form at 3 h after SE (Fig. 6*E*; control, 100.0 ± 7.9 vs SE, 85.0 ± 2.6; *t* test, *p* > 0.05); however, there is a significant reduction at 24 h after SE (Fig. 6*F*; control, 100.0 ± 5.0 vs SE, 65.4 ± 4.1; *t* test, *p* < 0.01). Most notably, there is a robust increase in the immunoreactivity of PAI-1, an inhibitor of both furin and tPA, at 3 h after SE (Fig. 6*G*; control, 100.0 ± 13.1 vs SE, 183.8 ± 18.2; *t* test, *p* < 0.01) and 24 h after SE (Fig. 6*H*; control, 100.0 ± 12.3 vs SE, 590.8 ± 63.3; *t* test, *p* < 0.001).

**Figure 7.**
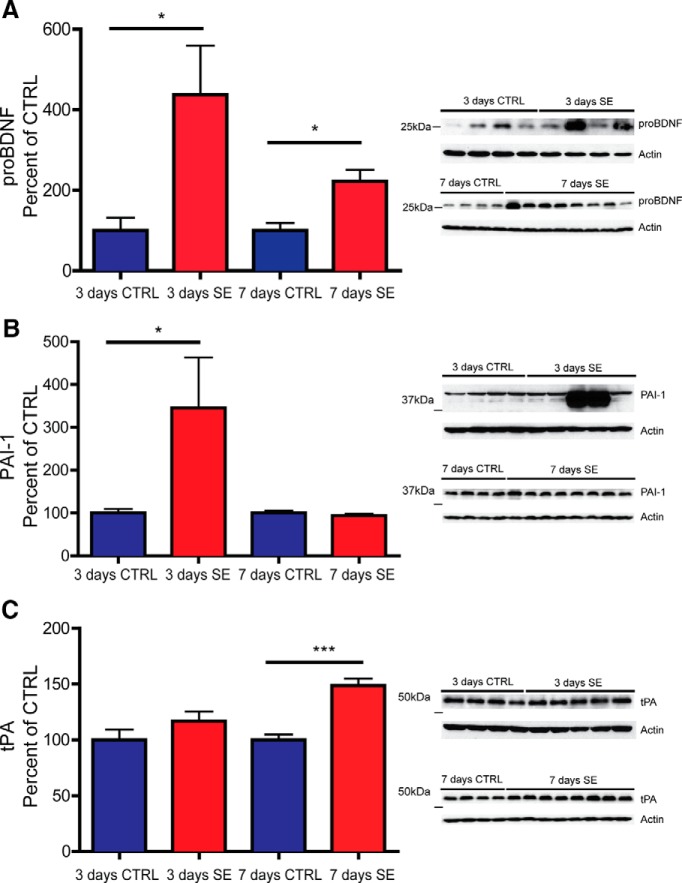
ProBDNF, PAI-1, and tPA levels at 3 and 7 d following SE. ***A–C***, Right, Representative Western blots of whole hippocampal protein homogenates from WT mice killed 3 d (top panels) or 7 d (bottom panels) after the induction of SE or time-matched saline controls probed with antibodies against proBDNF (***A***), PAI-1 (***B***), or tPA (***C***). Left, Densitometry analysis of abundance of proBDNF (***A***), PAI-1 (***B***), or tPA (***C***) normalized to actin and expressed as the percentage change relative to mean values of control group (±SEM). Anti-proBDNF (1:2000; ***A***); anti-PAI-1 (1:1000; ***B***); and anti-tPA (1:11,000; ***C***). *N* = 4 for all control groups, and *N* = 8 for all 7 d SE groups. For 3 d SE groups, *N* = 4 for proBDNF and *N* = 5 for PAI-1 and tPA (*p < 0.05, ***p < 0.001; *t* test was used for all analyses except PAI at 3 d, for which the Mann–Whitney (nonparametric) test was used due to a non-normal dataset).

To determine whether changes in the levels of proBDNF, PAI-1, and tPA persisted beyond 24 h after SE, levels of these proteins were examined at 3 and 7 d following SE in wild-type C57BL/6J mice. As can be seen in Figure 7*A*, the mean proBDNF level appears to peak at 3 d following SE, although there is more variability at this time point than at earlier time points (Fig. 7*A*; control, 100.0 ± 31.8 vs SE, 437.0 ± 122.0; *t* test, *p* < 0.05). At 7 d post-SE, proBDNF levels remain elevated, but the relative increase versus control is less than at 3 d and variability is lower (Fig. 7*A*; control, 100.0 ± 18.8 vs SE, 222.0 ± 28.5; *t* test, *p* < 0.05). PAI-1 levels are variable but overall remain elevated at 3 d (Fig. 7*B*; control, 100.0 ± 9.4 vs SE, 345.0 ± 117.8; Mann–Whitney test, *p* < 0.05), and have returned to control levels at 7 d (Fig. 7*B*; control, 100.0 ± 5.8 vs SE, 94.0 ± 4.0; *t* test, *p* > 0.05). Of note, the same two samples showed very high levels of proBDNF and PAI-1, and, interestingly, these two samples both came from animals that had extremely severe SE (multiple stage six seizures). Levels of tPA were not different from controls at 3 d after SE (Fig. 7*C*; control, 100.0 ± 9.2 vs SE, 116.0 ± 8.6; *t* test, *p* > 0.05), and were elevated compared with controls at 7 d after SE (Fig. 7*C*; control, 100.0 ± 5.0 vs SE, 149.0± 6.0; *t* test, *p* < 0.001).

**Figure 8.**
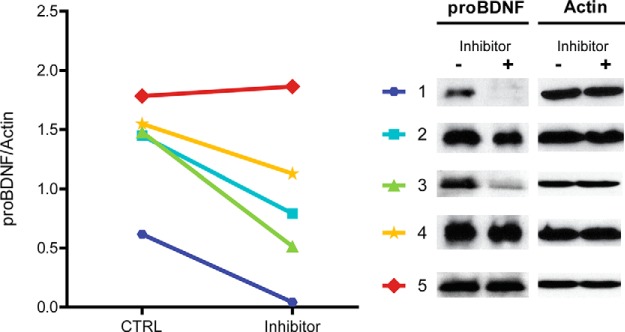
PAI-1 Inhibition reduces proBDNF levels after pilocarpine SE. Right, Representative Western blots of protein homogenates from hippocampal slices from individual WT mice removed 24 h after SE then incubated for 4 h in aCSF containing the PAI-1 inhibitor tiplaxtinin (370 μm; Inhibitor +) or vehicle (DMSO; Inhibitor −), probed with anti-proBDNF (1:2000) or anti-actin antibodies. Left, Densitometry analysis of abundance of proBDNF normalized to actin in homogenates from vehicle-treated (CTRL) and tiplaxtinin-treated (Inhibitor) slices for each animal (*N* = 5). Tiplaxtinin treatment resulted in a significant reduction in proBDNF levels compared with vehicle treatment (*p* < 0.05, *t* test).

To better assess whether there was a causal relationship between elevated PAI-1 levels and increased proBDNF levels after SE, we examined the effect of PAI-1 inhibition on proBDNF levels in hippocampal slices from wild-type C57BL/6J mice 24 h after SE. Hippocampal slices were rapidly removed and incubated for 4 h in aCSF containing the PAI-1 inhibitor tiplaxtinin (370 μm) or vehicle (DMSO). Tiplaxtinin treatment resulted in a significant reduction in proBDNF levels compared with vehicle treatment in slices from four of the five animals (Fig. 8; *p* < 0.05, paired two-tailed *t* test), suggesting that a reduction in proBDNF cleavage due to increased PAI-1 may be contributing to the elevation of proBDNF levels acutely after SE.

## Discussion

These studies provide evidence for an increase in the levels of proBDNF acutely following the induction of SE. In WT C57BL/6J mice as well as in HA epitope-tagged BDNF knock-in C57BL/6J mice, there is an increase in proBDNF as early as 3 h after SE onset, with levels remaining elevated at 24 h and peaking at 3 d post-SE. The epitope-tagged knock-in C57BL/6J mice were further used to localize early increases in BDNF after SE and HA immunoreactivity was detected primarily in principal cells but also some astrocytes throughout all hippocampal subfields. Finally, we demonstrated that acute increases in proBDNF after SE are associated with changes in the enzymes involved in the proteolytic processing of proBDNF (reduced tPA and increased PAI-1), and that enhancing proBDNF cleavage by inhibiting PAI-1 reduces proBDNF levels in hippocampal slices from animals 24 h after SE. Together, these results suggest that the inhibition of proBDNF cleavage contributes to acute elevations of proBDNF within hours of SE onset, positioning proBDNF to participate in early cell-signaling events after SE, such as activation of the JAK/STAT pathway.

Several groups have previously evaluated the levels of BDNF in epilepsy models (Ernfors et al., 1991; Rudge et al., 1998; Altar et al., 2004; Roberts et al., 2006). The majority of these studies quantitatively evaluated levels of mRNA, but did not determine the levels of proBDNF and mBDNF protein. A few studies have evaluated the levels of proBDNF and mBDNF after seizures, but none have reported an increase in proBDNF <24 h after SE onset. Unsain et al. (2008) analyzed the levels of BDNF following pilocarpine-induced SE in adult rats and demonstrated an increase in proBDNF immunoreactivity 3 d after SE. Using immunohistochemistry, Volosin et al. (2008) reported an increase in proBDNF immunoreactivity 1 d after pilocarpine-induced SE in rats. Elevated levels of proNGF protein have also been observed 24 h after kainate-induced seizures *in vivo* (Volosin et al., 2008; Le and Friedman, 2012). This increase in proNGF was not accompanied by increases in mature NGF and resulted from inhibition of MMP-7 by TIMP-1 (Le and Friedman, 2012). Most recently, it was reported that both proBDNF and mBDNF levels were elevated 24 h after a single dose of pilocarpine SE in 129SvJ mice (VonDran et al., 2014).

There may be a number of potential reasons why we were able to identify increases in proBDNF earlier after SE than had been previously reported, including differences in the species (rats vs mice), background strain (129SvJ vs. C57BL/6J), and model of SE (kainate or single high-dose pilocarpine vs repeated low-dose pilocarpine). Repeating the studies using the same techniques on different models would help to determine the effect of model selection on the findings. Another distinction between the previous studies and the one presented here is the use of BDNF epitope-tagged knock-in mice that allowed the levels of BDNF protein to be probed with high sensitivity and specificity.

To delineate the spatial expression of BDNF in response to SE, immunohistochemistry for HA was combined with MAP2 and GFAP staining in BDNF-HA-tagged mice to evaluate expression in neurons and astrocytes, respectively. The pattern of immunoreactivity for total BDNF in controls is similar to what has been previously reported by others (Dieni et al., 2012; VonDran et al., 2014). BDNF immunoreactivity is increased 3 h after SE, and colocalizes with MAP2 and GFAP, demonstrating that acutely following SE proBDNF is expressed in principal neurons and astrocytes in all hippocampal subfields. BDNF immunoreactivity is most strikingly elevated in the cell bodies of principal neurons of CA3 and CA1 and the mossy fiber pathway. Unfortunately, one is unable to determine whether the BDNF localization corresponds with a site of prerelease or internalization. One possibility to explain the presence of BDNF immunoreactivity in non-neuronal cells is that the BDNF localized in the astrocytes may be due to internalization, since TrkB.T1 is located primarily in hippocampal astrocytes. The truncated Trk receptors can function as a dominant-negative inhibitor by forming heterodimers with full-length TrkB leading to internalization of BDNF and triggering clearance of BDNF and TrkB (Haapasalo et al., 2002).

To identify potential mechanisms leading to rapid elevations in proBDNF after SE, we examined the expression of cleavage machinery known to process proBDNF into mBDNF. These studies were based on the hypothesis that the observed rapid elevation in proBDNF levels may be due, at least in part, to a reduction in proneurotrophin cleavage, akin to the inhibition of MMP-7 cleavage of proNGF reported to occur after kainate-induced SE in rats (Le and Friedman, 2012). Two potential mechanisms that could contribute to reduced proBDNF cleavage were identified: a significant decrease in tPA levels and a robust increase in PAI-1 levels. The tPA/plasmin proteolytic machinery is a major contributor to extracellular proBDNF cleavage, and PAI-1 inhibits both furin and tPA, thereby inhibiting both intracellular and extracellular cleavage of proBDNF. Elevation in the levels of PAI-1 normally corresponds to depression in tPA activity. It is thought that elevation of PAI-1 is an adaptive mechanism to attenuate excessive tPA activity, which can contribute to CNS pathology (Melchor and Strickland, 2005). In addition, theta burst stimulation (14,400 pulses, 60 min) triggers the simultaneous release of proBDNF and tPA to generate mBDNF extracellularly *in vitro*, and exogenous administration of PAI-1 inhibited tPA activity and attenuated the conversion of proBDNF into mBDNF (Nagappan et al., 2009). Together with our data demonstrating that PAI-1 inhibition with tiplaxtinin reduces proBDNF levels in hippocampal slices from mice 24 h after SE, these findings suggest that PAI-1 is a major regulator of proBDNF conversion to mBDNF under both normal physiological conditions and following seizures.

A limitation of the current study is that we were unable to fully evaluate mBDNF levels in parallel with the changes in proBDNF. Unfortunately, in our hands significant intralot variability and poor specificity were observed with commercial antibodies that reportedly identify mBDNF, including the antibody used in the recent report by VonDran et al. (2014), and therefore studies using mBDNF antibodies were not included in this report. Although we were able to identify clear and specific bands at the reported sizes of both proBDNF and mBDNF on Western blots of protein lysates from BDNF-HA-tagged mice reacted with an anti-HA antibody, due to difficulty in breeding we had only sufficient numbers of these mice to examine the earliest time points (3 and 24 h). Therefore, although we did not find evidence of an increase in mBDNF at these early time points after SE, we were unable to confirm this with a secondary method (using mBDNF antibody detection) and could not evaluate whether mBDNF elevations occurred at later time points after SE. An additional potential concern in using BDNF-HA-tagged mice to follow the endogenous presence of BDNF is the possibility that the HA tag may alter BDNF expression and/or processing. This seems unlikely, however, as it has been previously reported that the HA-tagged BDNF is expressed, processed, and trafficked in the same manner as WT BDNF (Yang et al., 2009, 2014).

The current study also does not investigate the downstream consequences of elevated proBDNF levels following SE. The rapid increase in proBDNF after the onset of SE is temporally positioned to mediate BDNF-induced JAK/STAT activation that begins within an hour of SE onset (Lund et al., 2008). Interestingly, high-dose proBDNF has recently been reported to lead to repression of GABA_A_R synthesis in cultured hippocampal neurons *in vitro* through activation of the JAK–STAT–ICER pathway (Riffault et al., 2014), suggesting that it might also mediate JAK/STAT activation and subsequent GABA_A_R α1 subunit repression *in vivo* after SE. Thus, further studies are required to fully understand the downstream molecular effects of the acute elevation in proBDNF levels demonstrated in these studies and the role of proBDNF signaling in epileptogenesis.

In summary, we demonstrate an increase in proBDNF levels that occur as early as 3 h after SE in principal neurons and their processes, as well as in astrocytes, throughout all hippocampal subfields. We further present evidence that the elevation in proBDNF is due, at least in part, to reductions in proBDNF cleavage that result from acute decreases in tPA expression and increases in PAI-1, an inhibitor of both intracellular and extracellular proBDNF cleavage. These findings suggest that proBDNF is highly abundant immediately following SE onset and may be a key component of neurotrophin signaling during the earliest phases of epileptogenesis.

## Synthesis

The decision was a result of the Reviewing Editor Tallie Z. Baram and the peer reviewers coming together and discussing their recommendations until a consensus was reached. A fact-based synthesis statement explaining their decision and outlining what is needed to prepare a revision is listed below. The following reviewer(s) agreed to reveal their identity: Celine Dube;

The Reviewers identified remaining, relatively minor issues that need to be addressed in the revision:

A new experiment that has been added to the revised manuscript, shows that treating tissue from SE animals with an inhibitor of PAI-1 reduces proBDNF suggesting that PAI-1 may play a role in SE-induced proBDNF; given the limitations of this experiment though it is difficult to know the degree to which PAI-1 is directly involved mechanistically, or if simply inhibiting this protein restores normal proBDNF levels. Despite this point, the manuscript has been substantially revised to address the majority of the concerns from the previous review and is greatly improved. There are still some minor points that need to be addressed.

1. While it is appreciated that the authors were addressing a review comment, the detailed discussion on MAP2 (lines 406-423) seems excessive and out of place in the present work considering that the authors didn't quantify the results and only mention them in passing.

2. The methods for the immunocytochemistry results and the results on pg 13 (line 275) are confusion. The methods state that they did triple immune labeling (HA, MAP2, GFAP), but the results say either HA/MAP2 or HA/GFAP. The authors need to resolve this.

3. Is Figure 4C from control or SE? If this is SE, then the authors need to amended the statement that BDNF is localized to astrocytes "in SE animals" (Line 288).

4. Stats in both the figures and results should agree. Also, some of the stats incorrectly use "&gt;" rather than "&lt;"; lines 304 and 305.

5. Figure 5 caption needs p values for asterisks.

6. Results pg 15 second paragraph. Figure 7 panels should be referred to as A, B, C. The blot for figures 7 and 8 are exceedingly dark (over exposed?) as compared to the other blot figures in the manuscript.

7. Figure 7B, 3hour data. Did the authors use a non-parametric t-test?

8. Discussion. Line 356. Only the HA mice showed further rise in proBDNF where as the non-transgenic mice showed a relative reduction by 24h (∼300% at 3 hr, ∼175% at 24h). Because the two methods differ, the authors shouldn't use the blanket statement that there was a further increase; it was still elevated.

9. Line 393. "ir" is used for immunoreactivity in the Discussion, but it might be more useful to use it earlier in the paper.

10. The manuscript needs a thorough proof-reading; wording is repeated, dropped words, errors in citations, etc. All acronyms should be defined.
